# Medicines governance and systemic constraints: A qualitative study among healthcare professionals in South Africa

**DOI:** 10.4102/phcfm.v18i1.5356

**Published:** 2026-06-18

**Authors:** Miriam M. Ndwandwe, Mygirl P. Lowane, Thembi V. Simbeni, Mathildah M. Mokgatle

**Affiliations:** 1 Department of Public Health, School of Healthcare Sciences, Sefako Makgatho Health Sciences University, Pretoria, South Africa

**Keywords:** essential medicines, medicines governance, supply chain management, medicine shortages, Pharmacy and Therapeutics Committees, Standard Treatment Guidelines, health systems

## Abstract

**Background:**

Medicine shortages and governance failures in low- and middle-income countries continue to hinder progress towards universal health coverage.

**Aim:**

To explore healthcare professionals’ perceptions and experiences of medicines governance in South African healthcare facilities.

**Setting:**

The study was conducted at the public healthcare sectors in Eastern Cape province, South Africa.

**Methods:**

A qualitative exploratory cross-sectional design was used. Twenty healthcare professionals were purposively selected. Semi-structured interviews were conducted, audio–recorded, transcribed verbatim and thematically analysed using Braun and Clarke’s framework.

**Results:**

Four major themes emerged: systemic governance constraints; pharmaceutical supply chain vulnerabilities; weak accountability mechanisms; and gaps in protocol implementation. Key determinants of medicine shortages included weak budget governance, supplier payment delays, limited pharmacy support staff and inconsistent implementation of standard treatment guidelines (STG) and limited functionality of Pharmacy and Therapeutics Committees (PTCs).

**Conclusion:**

Medicine shortages result from systemic governance failures rather than isolated logistical issues. Strengthening pharmaceutical governance requires transparent budgeting, timely supplier payment systems, functional PTCs, and STG training to improve equitable access to essential medicines.

**Contribution:**

This study deepens the understanding of pharmaceutical governance, revealing systemic, interrelated factors that collectively lead to the shortage of pharmaceuticals in South Africa. Unlike previous research that focused primarily on supply chain logistics, the study showed that stock-outs were rooted not simply in operational inefficiency, but also in structural governance weaknesses. This study presents practical insights to strengthen pharmaceutical governance and supports national efforts to achieve universal health coverage by identifying specific governance obstacles and proposing system-level reforms.

## Introduction

Healthcare progress has led to a steady rise in life expectancy and quality of life, significantly boosted by global economic growth.^1^ Access to essential medicines is a crucial component of the health system to ensure that people receive the necessary treatments for disease prevention, diagnosis and treatment.^2^ In low and middle-income countries, fragmented supply chains and inflexible resource allocation processes impede timely, affordable delivery of important medicines and continue to be a global health concern.^3,4^

Because the term ‘access’ is complex, there is no definition of ‘access to essential medicines’ that is universally agreed upon. However, access is described as the ability to obtain necessary medicines at reasonable prices and on time.^5^ In the past 50 years, the concept of essential medicines has changed. The World Health Organization’s (WHO) list of essential medicines (EML) has evolved over time as a result of the widening scope and financial complexity to determine which medicines are considered essential.^6^ The cost and accessibility of essential medicines within the health system, including local and private pharmacies, were found to influence their availability.^7^ Moreover, the inability to pay for medication may be due to the client not being insured and a shortage of drugs at service delivery locations.^8^ The public’s access to medicine is dependent on either out-of-pocket expenses or National Health Insurance (NHI) coverage.^9,10,11^

One of the most urgent issues facing patients and healthcare systems in high-, middle-, and low-income nations is the high cost and scarcity of medications.^12^ The correct assessment of the availability is further hampered by the disregard for the patent status and quality of medications.^13^ Ethically, people should not be refused access to life-saving or health-promoting interventions for unfair reasons, such as social or economic gain. The main goal is to ensure that medicine availability is guaranteed.^14,15^

Eighteen of the world’s poorest nations are found in sub-Saharan Africa (eastern, western, central, and southern Africa), where at least 40% of people live below the global poverty threshold. As a result, the area has poor health systems, a high disease burden and a lower life expectancy of 64.5 years versus the global average of 73 years. In addition, malaria, human immunodeficiency virus (HIV) and/or acquired immunodeficiency syndrome (AIDS), tuberculosis (TB), lower respiratory tract infections, diarrhoea and other communicable (infectious) diseases account for more than 53% of all recorded fatalities in the region and dominate morbidity and mortality.^16^

The equitable and continuous availability of essential medicines remains a fundamental goal of health systems worldwide.^8^ However, persistent medicine shortages and governance failures in low- and middle-income countries (LMICs) continue to threaten the realisation of universal health coverage.^3^ Medicines governance refers to the rules, procedures, and frameworks that ensure decisions about the procurement, distribution, and use of pharmaceuticals are transparent, accountable, and aligned with clinical priorities.^17^ Governance challenges in South Africa’s pharmaceutical system, such as fragmented decision-making, budget misalignment and weak oversight, contribute to medicine shortages, especially in under-resourced provinces.^18^ Poor communication among financial and operational managers, along with a shortage of human resources, were identified as persistent challenges in implementing the pharmaceutical governance system, which encompasses the policies, processes and oversight mechanisms that guide the selection, procurement, distribution and use of medicines within the health system.

Medicine stock-outs and delayed procurement affect all nine provinces in South Africa and, consequently, the service delivery.^19,20,21^ With this identified gap, this study aimed to explore healthcare professionals’ perceptions and experiences of medicine governance in healthcare facilities. The study will helpfully address constraints in the health system, especially by promoting better decision-making and clarifying the implications of patient care.

## Research methods and design

### Study design

An exploratory descriptive qualitative design was adopted to obtain in-depth information from the study participants. This produced a broader description of participants’ views and experiences, and the meanings of their perceptions were analysed. The study explored perceived experiences of healthcare professionals to gain more information regarding medicine governance in healthcare facilities.

### Study setting

The study was conducted in one of the South African provinces. This province is one of the largest provinces in South Africa, situated along the southeastern coast. It is predominantly rural, and isiXhosa is the most widely spoken language. The province has a two-tier health care system: the public and private health sectors. Those health sectors primarily offer comprehensive primary and secondary health care services. The province faces shortages of physicians, pharmacists, as well as inadequate infrastructure, especially in the public healthcare facilities in rural settings. Mobile services play an essential role in reaching remote communities, supporting the provincial government’s goal of promoting health through primary health care, strengthening the health system, and preparing for the implementation of National Health Insurance (NHI). The province’s health system is facing governance challenges, periodic medication shortages and systemic resource constraints. The study setting also includes Pharmacy and Therapeutics Committees (PTCs), which are multidisciplinary bodies responsible for overseeing the selection and rational use of medicines, developing and maintaining formularies, and providing guidance on medicine procurement and use within the healthcare system.

### Study population and sampling

The study population comprised healthcare professionals occupying managerial roles with responsibility for medicines management and governance within public healthcare facilities in South Africa. These included pharmacists, medical officers and professional nurses who held formally designated supervisory or managerial positions at the level of clinics, community health centres (CHCs), hospitals and provincial health offices.

Participants were eligible for inclusion if they were directly involved in medicines governance processes, including decision-making, oversight and implementation of medicines-related policies and practices. Individuals in exclusively administrative or non-clinical roles, such as those in finance or supply chain management, were excluded, as they were not directly engaged in clinical or medicines governance functions. A sample size of approximately 15–25 participants was anticipated, based on qualitative research guidance for achieving depth and thematic saturation.^22^ The final sample size of 20 participants was determined by data saturation, defined as the point at which no new themes or insights emerged from successive interviews. Recruitment ceased once redundancy in the data was observed.

A purposive, criterion-based sampling strategy was used to select participants with direct experience in medicines governance.^23^ Selection criteria included: (1) professional role (pharmacists, medical officers and professional nurses), (2) involvement in medicines management and decision-making processes, and (3) representation across different levels of care (primary, secondary and tertiary) and health system structures (facility, district and provincial levels). This approach ensured diversity in professional perspectives, levels of responsibility and healthcare contexts.

### Data collection process

Participant recruitment was conducted in person by the primary investigator. Upon obtaining ethical clearance approval and permission from the province, the researcher visited all the healthcare facilities to arrange appointments with eligible, interested individuals. The appointments to meet with the facility managers were arranged via the managers’ personal assistants. At primary healthcare facilities without personal assistants, the researcher arranged appointments directly with operational managers. On the day of the appointment, the researcher explained the purpose of the study before seeking their consent to participate. For those who consented, further arrangements for interviews were made.

Data were collected using a semi-structured interview guide which was developed based on the findings of the quantitative phase (Phase One) of the primary researcher’s Doctor of Philosophy (PhD) project. The questions were specifically derived from key quantitative results that required further exploration, in-depth explanation and clarification. The interview guide was organised into distinct sections. Section A of the interview guide captured participants’ socio-demographic and professional characteristics. Section B comprised the main interview component which was led by the following open-ended question: *Can you describe your experiences and perceptions of medicines governance in your facility?*

This opening question was designed to elicit broad, reflective responses, after which targeted probing questions were used to explore emerging issues in greater depth.^24,25^ To guide the data collection process and the flow of conversation, a semi-structured interview guide with sets of open-ended and probing questions was used throughout the interviews. The following were the key topics covered in the interview guide:

- Communication processes in medicines budget allocation within healthcare facilities.
- Roles and responsibilities of healthcare professionals in medicines stock management.
- Decision-making factors and stakeholders involved in annual medicines budget allocation.
- Impact of poor governance and medicines stock-outs on healthcare professionals.
- Communication and monitoring of compliance with Standard Treatment Guidelines (STGs) among newly appointed clinical staff.
- Healthcare professionals’ involvement in PTCs.

Upon securing appointments with the participants who were healthcare professionals and managers, individual interviews were conducted at their workplace in English. Interviews were conducted in the morning before participants began their daily routine, while others opted to be interviewed during lunch time and shortly after working hours. The interviews were conducted in the managers’ offices.

Data were collected between July 2025 and October 2025, with one interview per day and three interviews per week. Due to the complexity of the study, each interview lasted 45 min to 90 min. All interviews were recorded with a voice recorder with the permission of the participants. Field notes were taken in the course of the interview and recorded immediately after the interview to capture contextual details, non-verbal clues and reflective observations. These notes complemented interview data and enhanced interpretations by providing additional context to the interview transcripts.

A pilot phase was conducted with three individuals who met the study inclusion criteria to assess the feasibility of the data collection process and the suitability of the semi-structured interview guide. The pilot interviews enabled the researcher to evaluate the clarity, flow and relevance of the interview topics, as well as the overall conduct of the interviews. Based on insights gained during this phase, minor revisions were made to the wording and sequencing of selected prompts to enhance clarity and facilitate more in-depth responses. The pilot also informed the refinement of probing techniques and interview approach. Data generated during the pilot interviews were included in the final analysis, as participants’ contributions were relevant to the study objectives.

### Data analysis

Braun and Clarke’s six-phase thematic analysis framework^26^ was used to guide qualitative interview analysis. The framework is particularly suitable for this study because it provides a clear and systematic process for identifying, analysing and reporting meaningful data patterns. A thematic analysis with a deductive approach was employed.^27^ The audio-recorded data were transcribed verbatim. Transcripts were cleaned, proofread and arranged for analysis.

Two researchers, who were the lead (first) author and the study supervisor (second author), were involved in the coding process. The same transcripts were independently double-coded by both researchers (i.e., transcripts were not split between coders) to ensure consistency and enhance the credibility of the analysis. An independent coder was also engaged to further strengthen the rigour of the coding process. Following independent coding, the researchers and the independent coder met to compare coding, discuss discrepancies and reach consensus on the interpretation of the data. Through this iterative process, a codebook was developed and refined, with clear definitions of codes, themes and sub-themes. The lead researcher managed the project file for NVivo (version 14) by importing transcripts and data encodings into the software to facilitate data organisation. The NVivo project file was shared with fellow researchers to enhance the rigour and credibility of the analysis.

### Measures of trustworthiness

Lincoln and Guba’s (1985) four pillars for establishing reliability in qualitative research – credibility, dependability, confirmability and transferability – were adopted.^28^ Credibility was strengthened by extended field engagement, triangulation, member checking and peer debriefing. The triangulation of the data sources further strengthened the credibility by obtaining perspectives from different types of health professionals, including pharmacists, medical officials and professional nurses. Member checking was conducted by returning interview transcripts to participants to verify the accuracy and completeness of the captured information. Participants were given the opportunity to review their responses and confirm that the transcripts were a true reflection of their views. Lastly, peer debriefing was conducted among all the authors to review and refine the study findings.

Social desirability effects were reduced by conducting interviews in the participants’ offices and assuring them that the study findings would not be linked to their identities. This made them feel comfortable with the fact that they were recording their experiences and perceptions of the management of medicine in their context. The guarantee of confidentiality and the creation of a comfortable environment encourage honest responses.

To ensure dependability, an audit trail was maintained by recording all stages of the research process, from data collection and code decisions to theme development and the reporting of findings. Moreover, the methods of this study were clearly outlined to enable other researchers to assess or replicate the study in a similar context.

To enhance confirmability, the lead researcher engaged in continuous reflection throughout the study. The researcher is a pharmacist trained in medicine governance and currently holds senior management positions in the public health system. This professional background not only provides in-depth knowledge of the research environment but also positions the researcher as an insider of the research environment. Researchers have professional relations with the topic, and in some cases have similar organisational environments with participants, but there are no direct managerial relationships. This positionality was crucial for awareness, as it can influence both the collection and interpretation of data.^29^ To mitigate potential bias, the researcher kept a reflection journal to document personal assumptions, preconceptions and evolving interpretations during the research process. In addition, interview techniques such as open-ended questions and neutral probing were used to minimise response bias. Ongoing discussions with the other researchers were also conducted to critically assess the emerging findings and to challenge the possibility of the subjective interpretations.

Transferability was enabled by providing a detailed and in-depth description of the study context, participant characteristics and research processes. This allows judgement on whether the results of this study can be applied in other studies of a similar context.

### Ethical considerations

Ethical clearance to conduct this study was obtained from Sefako Makgatho Health Sciences University Research Ethics Committee (No. SMUREC/H/28/2024:PG). Core ethical principles guided the study’s conduct. The Health Department of the Eastern Cape province granted the permission to carry out the study. Informed consent is a cornerstone of ethical research, ensuring that participants voluntarily agree to participate in a study with a clear understanding of what it entails. The researcher explained the study’s purpose, procedures and benefits in a way that is understandable to participants. This empowers participants to make an informed decision about their contribution, safeguarding their rights, dignity and autonomy. In this study, participants were informed of their right to decline participation or withdraw at any time without negative consequences. Confidentiality and privacy were maintained by not asking for their names during interviews and by using a number to code the transcripts. Non-maleficence requires researchers to protect the participants from intentional harm or inflicting unnecessary suffering. The researcher protected the participants from any psychological or physical harm by adhering to the research question and interviewing them in a safe place. Furthermore, the researcher also protected the participants’ views, as this study might create negative consequences for them at other levels. To ensure the principles of justice, the researcher asked all participants the same questions regardless of their positions in the workplace. Participants’ rights to privacy and personal data protection were maintained in accordance with the *Protection of Personal Information Act no. 4 of 2013* (POPIA).

## Results

### Demographic information of the study participants

A total of 20 health management executives from the management and professional categories of the health system participated in the study. Participants included operational managers, clinic managers, nurses and pharmacists, medical officials and senior administrators such as provincial coordinators, policy specialists and executive directors. The group was composed of seven pharmacists, six medical officers and seven nurses, and represented key areas of medical supplies, clinical services and policy management. Participants were between 33 years old and 56 years old, with an average age of 46 years. The number of years of service ranges from 8 years to 30 years, while the management experience ranges from 1.5 years to 18 years, indicating a group of experienced participants. With regard to gender distribution, 11 participants were men and 9 women. Table 1 presents the demographic data of the study participants.

**TABLE 1:** Demographic data of the participants.

Participant number	Professional category	Gender	Age (years)	Years of service	Years as managers
1	Nurse	F	47	25	10
2	Pharmacist	M	38	14	5
3	Medical officer	M	35	9	2
4	Nurse	F	42	19	7
5	Pharmacist	M	50	25	15
6	Nurse	F	53	29	14
7	Medical officer	M	33	8	1.5
8	Pharmacist	F	41	17	6
9	Nurse	F	44	20	9
10	Medical officer	M	49	23	10
11	Pharmacist	M	56	30	18
12	Pharmacist	F	45	21	10
13	Medical officer	M	51	25	12
14	Pharmacist	F	48	24	11
15	Medical officer	M	46	20	8
16	Medical officer	F	50	26	9
17	Medical officer	M	52	30	12
18	Pharmacist	M	43	19	6
19	Nurse	F	54	28	13
20	Pharmacist	M	49	22	11

F, female; M, male.

### Themes and sub-themes

Four themes and seven sub-themes emerged that illuminate the systemic governance and operational barriers influencing the availability of medicines in the healthcare system, as presented in Table 2.

**TABLE 2:** Themes and sub-themes.

Themes	Sub-themes
1: Systemic governance constraints	1.1 Budget governance and financial communication 1.2 What informs the allocation of the medicine budget
2: Pharmaceutical supply chain vulnerabilities	2.1 Stock and supply chain management 2.2 Supply chain weaknesses and procurement delays
3: Escalation of complaints and public accountability	3.1 Impact of poor governance and stock-outs
4: Protocol integrity versus system failure	4.1 Standard Treatment Guidelines (STG) communication and implementation 4.2 The functionality of Pharmacy and Therapeutics Committees (PTCs)

#### Theme 1: Systemic governance constraints

This theme includes governance issues affecting the management and access to medicines. Participants highlighted issues such as a lack of communication between the administrative and medical levels, financial control problems and inconsistencies in decision-making. Budgetary governance and financial communication are the basis for the distribution of medical budgets, which appear to be sub-themes that show how the lack of transparency and coordination reduces the distribution of resources and impedes the provision of effective services.

Sub-theme 1.1: Budget governance and financial communication: It is reported that, at all levels of management, the medical budget does not have transparency and consultation. Budget allocations are generally communicated only after the fact, and usually delivery of drugs is delayed or incomplete, making it difficult for facility managers to effectively plan. Participants in the primary health system have pointed out that budgetary decisions are ‘top down’ without feedback channels or official communications. The director of a clinic mentioned the following:

‘We don’t receive any formal communication when there are changes to the medicines budget … we usually find out indirectly.’ (P1, nurse, female, 47 years old)

Even senior managers and other officials echoed similar frustrations, showing that financial decisions prioritised cost reduction over clinical need.

One participant stated with some concerns:

‘By the time we are informed of the allocation, the decision is final and is not open to discussion.’ (P2, pharmacist, male, 39 years old)

One executive manager remarked:

‘Decisions are often made in response to fiscal pressures rather than clinical need.’ (P10, medical officer, male, 49 years old)

Sub-theme 1.2: Allocation of the medicine budget is not aligned with clinical trends and service needs: Respondents at all levels of the administration questioned the transparency and rationale behind the allocation of the medical budget. Although hospitals regularly publish monthly statistics on the number of patients and their drug consumption, many believe that this information has little to do with the final budgetary decision. Instead of reflecting changing clinical needs, budget allocations seem to be based on previous expenditures.

One clinic manager said the following:

‘We are filing a monthly report, but the way this data is used is unclear. When we began to see more diabetes cases, I expected the number to rise, but that has never happened.’ (P9, nurse, female, 44 years old)

Several participants noted that funding decisions rarely reflected population growth, service expansion and new disease patterns. A nursing manager said:

‘The following year, we opened a new paediatric wing and received the same medical budget.’ (P6, nurse, female, 53 years old)

Overall, participants indicated that facility data and state budgeting processes operate independently, with limited consideration given to clinical trends, service complexity, and Standard Treatment Guidelines (STGs) in financial planning.

One participant pointed out that:

‘If STGs are not integrated into the cost planning and procurement cycles, they are only aspirational documents.’ (P17, medical officer, male, 52 years old)

#### Theme 2: Pharmaceutical supply chain vulnerabilities

This theme deals with operational and logistical problems that interfere with drug supply. The participants discussed the impact of supplier accounts suspension, delivery delays and systemic shortcomings in stock management. In general, participants noted that these problems showed that the distribution and procurement systems were weak and that they could not meet the needs of the facilities. The sub-themes of account suspension and stock and supply chain management show how supplier-related issues and administrative inefficiency undermine the reliability of the pharmaceutical supply chain.

Sub-theme 2.1: Stock and supply chain management: The participants often described stock management as a crisis-focused process rather than a stable, well-coordinated system. Clinics and CHCs have been mostly affected by these difficulties. Many facilities still use manual systems such as bin cards, often without the help of trained pharmacy assistants. Nurses and doctors in hospitals were forced to do pharmaceutical tasks with little or no formal training, which made them susceptible to errors, stress and fatigue. Here are some of their comments:

‘As a manager, I am responsible for ordering, receiving and recording inventory … Because of the shortage of pharmacy support staff, I often rely on my professional nurses … we sometimes refer patients to the nearest CHC or share doses, which is not ideal.’ (P19, nurse, female, 54 years old)

‘I had to play a role beyond the scope of my clinical work. I monitor bin cards, track large-scale medications and even follow up with the storage. It is an interim system. There is no pharmacy assistant, no IT system, only handwritten records and hope that the depot will deliver.’ (P7, medical officer, male, 33 years old)

Sub-theme 2.2: Supply chain weaknesses and procurement delays: Participants noted that, in most cases, health facilities were unaware that the pharmaceutical depot had not paid the supplier and came to know this only when they called the supplier to check with their orders. Some expressed their frustration that their facilities have already exhausted all the buffer stock in their shelves to sustain patient care. Furthermore, failure to settle suppliers’ accounts results in the complete depletion of medicine stock at health facilities.

A pharmacist shared:

‘When suppliers suspend our account, we must borrow from others to avoid patient crises.’ (P12, pharmacist, male, 45 years old)

‘My job is to ensure that the suppliers are paid, and the stock is delivered. If the department fails to pay the invoice in time, the contracted supplier suspends the delivery, and I am the victim of the failure.’ (P20, pharmacist, male, 49 years old)

‘Every delay in essential medicine can be fatal. Sometimes I had to work directly with the suppliers to ask for emergency stock while on the other hand request the Treasury to process the payments.’ (P13, medical officer, male, 51 years old)

#### Theme 3: Escalation of complaints and public accountability

This theme reflects the participants’ concern about the lack of response systems and a regulatory framework for drug shortages. Public confidence in the health system is threatened by increased complaints, often limited feedback, and delays in correction. The impact of poor governance and stock-outs is the sub-theme under this theme, underscoring the fact that a lack of clear accountability mechanisms exacerbated service interruptions and threatened patient care.

Sub-theme 3.1: The impact of poor governance and stock-outs: Participants at all levels of management have stated that shortages of medical supplies are one of the most painful consequences of bad governance, and this had a direct impact on patient care and the morale of the healthcare staff. Some participants emphasised that delays in medicines supply, treatment delays, and treatment adjustment were not only practical challenges but also matters of ethical and professional responsibility.

One nurse explained:

‘Staff morale has changed … Relations with the community are tense – they think we are incompetent.’ (P4, nurse, female, 42 years old)

Two participants reported that the shortage of medicines often leads to unsafe replacements, treatment interruptions and patient distrust by stating that clinicians are frustrated and begin to seek clinically unsafe alternatives and that the ethical burden on staff is enormous.

Medicine stock-outs have an adverse impact on the functioning of oncology, maternity and emergency care. This was highlighted by one participant:

‘We had a woman with PPH without ergometrin … the delay almost cost her life, recalls an operational director.’ (P9, nurse, female, 44 years old)

Some participants associated these deficiencies with systemic financial rigidity that contributed to accounting suspensions, procurement delays, preventable deaths, and rising medical claims. Several participants argued that the impact of budgetary bottlenecks reflected governance failures rather than clinical negligence, highlighting the responsibility of senior managers to account for such errors.

#### Theme 4: Protocol integrity versus system failure

Pharmaceuticals and Therapeutics Committee and STG have been established to ensure the rational use of drugs, but participants have reported inconsistent communication and poor compliance. The topics on STG and implementation relate to the gaps between the implementation of established treatment protocols and the supporting health systems. The sub-theme on the functionality of the PTC highlights structural and administrative weaknesses that undermine compliance with clinical protocols, fragment practice and compromise patient outcomes.

Sub-theme 4.1: Standard treatment guidelines and implementation: Participants noted that STGs were largely ineffective and inconsistent across all levels of the health system. Some also reported that many institutions lacked formal procedures for the induction of new employees and ensuring ongoing compliance, especially at the primary health care (PHC) levels:

‘When new employee is appointed, there is no structured orientation process. We cannot do the best if there is no formal orientation, perhaps a day-induction to discuss the use of the Essential Drug List [*EML*], though it’s not enough. I think people think everyone knows what STG is, but those who are newly employed lack knowledge on the basic principles outlined in the STGs, and it’s a risk.’ (P1, nurse, female, 47 years old)

According to the respondents, although some hospitals offered departmental or quarterly STG training, primarily for interns or community service physicians, uptake was uneven, and monitoring was limited due to workload constraints. Structured workshops had been discontinued in several facilities due to budget cuts:

‘We conduct STG training quarterly, mainly for interns and new community service doctors. But uptake is inconsistent. Some departments engage actively, others not. Compliance is monitored via prescription audits, but they are not frequent due to workload.’ (P14, pharmacist, female, 48 years old)

‘The training is informal. We sometimes discuss them [*STGs*] during the ward rounds, but there’s no structured orientation. Many junior doctors just do what the seniors do. As a results, it creates inconsistency. I only authorise orders for medicines that are on the PHC EML/STG, but there are instances where one finds a hospital-level medicines on our shelves.’ (P3, medical officer, male, 35 years old)

Sub-theme 4.2: The functionality of Pharmacy and Therapeutics Committees: The way PTCs operate varies greatly throughout the health system, according to the respondents. They mentioned that PTCs were typically active, multidisciplinary and powerful at tertiary and regional hospitals, assisting in the management of expensive medications, the rationalisation of formularies and the reduction of irrational prescribing. Some participants added that these committees were seen as useful governance tools that support the use of medicines in an economical, evidence-based manner:

‘Senior pharmacists from depots and districts, clinical programme managers, medical specialists, and representatives from finance make up the PTC at the province level. In addition to offering advice on sensible medication usage and stock prioritising, the PTC evaluates and approves changes to the provincial formulary. Making sure PTC decisions are communicated and executed at the facility level is a challenge.’ (P5, pharmacist, male, 50 years old)

On the other hand, participants reported that PTCs were frequently dormant and inadequately institutionalised at the PHC and CHC levels. Some of them highlighted that meetings and participation were irregular, and that there was little higher-level feedback:

‘We do not have a committee on drugs and therapy at the clinic level. I have only heard of it being active at the district or hospital level. Apparently, pharmacists, medical officials, programme managers and financial representatives are included. We have never received feedback from District PTC. I assume they are gathered, but we are not informed of what is being discussed.’ (P1, nurse, female, 47 years old)

Respondents from provinces and districts highlighted that although PTCs have made strong clinical and policy recommendations, procurement bottlenecks, budgetary limitations and poor communication throughout the system often make implementation difficult. Most respondents said that without specific funding and decision-making power, PTCs could become advisory organisations with little real influence.

## Discussion

The results showed the complex interplay between system governance constraints, supply chain vulnerability, erosion of responsibility, formal protocol and operational realities, which reduced the reliable availability of essential medicines. The study identified systemic governance challenges, financial communication gaps, opaque budgetary processes and limited coordination between clinical needs and resource allocation as key factors influencing the availability of medical supplies. These results strongly matched existing literature, showing that the challenges associated with the availability of medicines in LMICs were not due to logistical constraints, but a result of structural deficiencies in governance, funding and surveillance.^18,30,31^ As in the case of South Africa, Mauritania and Indonesia, participants pointed out that budgetary decisions are not based on service needs, but based on budgetary factors, thereby weakening the capacity of facilities to plan and respond to population health trends.^32,33^ Therefore, health governance reforms must give priority to a transparent and need-based budget integrated into the routine clinical data system to ensure that resource allocation practices meet the growing burden of diseases.

A lack of transparency in communication about medicines budgets and delays in payments to suppliers have led to a cyclical effect from operational disruptions to ethical difficulties among health professionals. The exclusion of the voices of healthcare staff from budgetary activities perpetuates decisions that are not in line with patient needs, while disintegrated procurement structures impede accountability. The experiences of the healthcare professionals show the moral damage caused by systemic failures: physicians and managers internalise system failures as their personal guilt. To restore resilience, the medicines governance reforms should give priority to a comprehensive financial planning, timely payments to suppliers and real-time stock data monitoring through integrated electronic information systems.

Another important finding highlighted the major weaknesses in the supply chain, particularly payment delays to suppliers and account suspensions. This confirms previous reports that bottlenecks in procurement and inefficient financial processes are the main determinants of stock flows in the public health sector in South Africa.^20,21^ Similarly, studies conducted in low- and middle-income countries (LMICs) have shown that weaknesses in financial governance undermine supplier relationships and disrupt the flow of medicines throughout the health system.^4,3^ Therefore, to strengthen supply chain resilience, continuous financial flows, timely payment of suppliers and simplified procurement management are essential to avoid service interruptions that can harm patient care.

Furthermore, this study revealed that the lack of medicines and governance inefficiencies have had a profound ethical and psychological impact on health workers. Participants described moral distress, staff morale decline, community distrust and pressure to compromise clinical standards when there were no essential drugs. Although previous research has documented the operational impact of medicine stock-outs, little is known about their emotional and ethical effects on front-line managers.^19^ This study adds to the literature showing how governance failures directly influenced the relationship between providers and patients and contributed to the disruption of services in health facilities. To protect the integrity of clinical practice, it is necessary to implement interventions not only in the administrative operations of healthcare facilities, but also in staff support systems and communication frameworks.

Another finding showed failure in the implementation of STGs and weaknesses in the functionality of PTCs, especially in primary care. This reflects similar challenges identified in Nigeria and South Africa’s PHC context, where the lack of structured training, inadequate direction and limited committee power undermine the rational use of drugs.^34,35^ Similar studies in Saudi Arabia and Ethiopia indicate that PTCs are often passively institutionalised because of limited resources and weak governance structures.^36,37^ The implication is that the strengthening of STG adoption requires structured and mandatory training, while reviving the Central Procurement Team (CPT) requires formal mandates, adequate resources and integration into the financial and procurement cycles.

Overall, the findings show a system of financial, managerial and operational failures that are interconnected and converge to cause a continuous shortage of medicines. This reflects the global evidence that single, fragmented governance models inhibit the reliability of the supply chain of essential medicines.^8,13^

### Strengths and limitations

Although this study has some limitations, it also has notable strengths. A key strength lies in the rich, in-depth qualitative data obtained from a diverse group of participants, including pharmacists, nurses, medical doctors and senior managers. This diversity enables a balanced understanding of governance challenges from the institutional perspective, often addressing important gaps in existing literature, focusing on supply chain data and policy analysis.^25^ The study also used rigorous, well-established methods to enhance the trustworthiness of the findings, such as member checking, triangulation and audit trails to increase the reliability of the results.^38^ This methodological robustness ensures that the interpretation reflects the real experience of the health system.

This study has some limitations. This study was conducted in a single province which limits the scope of the results beyond similar LMIC environments. This is consistent with the limitations stated in comparable qualitative studies on medicine governance.^31^ However, the study provides rich, contextually grounded insights that may be transferable to similar settings. The findings are likely to be relevant to other districts and provinces in South Africa, particularly those facing comparable governance and resource constraints. Furthermore, the systemic challenges identified such as weak financial governance, procurement delays and limited implementation of clinical guidelines are common across many LMICs, suggesting potential applicability beyond the study setting.

Although data saturation was achieved, with no new information emerging in the final interviews, it is possible that a broader or more diverse sample, particularly including stakeholders not represented in this study, may have yielded additional perspectives. Future research could include additional stakeholders, such as supply chain and finance personnel, to provide a more comprehensive understanding of decision-making processes and systemic constraints affecting medicine availability.

Despite efforts to minimise social desirability bias through assurances of confidentiality, some participants may have moderated their criticism of institutional structures. These limitations suggest that future research should adopt a multi-sectoral approach, incorporating perspectives from budget officials and other high-level decision-makers at both provincial and national levels who are responsible for allocating funds for essential medicines and determining procurement priorities. Such an approach would enable a more comprehensive assessment of medicine management systems.

### Implications

The implication is that the reform must take a system-thinking approach, integrate clinical, financial and logistical governance, achieve sustainable access to essential medicines and promote the commitments to universal health care.^14^ The results of this study have several implications. For the health system, it is necessary to strengthen governance structures by integrating clinical, financial and supply chain decision-making processes. For healthcare managers, improving transparency in budget allocation, ensuring timely payments to suppliers and strengthening accountability mechanisms are essential to preventing a shortage of drugs. For clinicians, systematic training and monitoring of compliance with STGs are essential to promote rational medicine use. For policymakers, the reactivation of the PTC with clear powers and resources could improve coordination across the system. Future research should take a multi-sectoral approach that incorporates perspectives from stakeholders in the finance and supply chain in order to better understand systemic constraints and inform policy-making.

## Conclusion

The study concluded that medicine shortages reflect deep systemic failures in management, financial oversight, supply chain operations and support for the rational use of medicines. The disconnect between clinical needs and budgetary processes, together with persistent procurement delays and the inconsistent implementation of monitoring systems such as Pharmacy and Therapeutics Committees (PTCs), collectively undermines the state’s ability to ensure continuous access to essential medicines. These results confirm that effective medical governance requires the integration of clinical data, financial decision-making, procurement efficiency, and treatment in accordance with global scientific research.

To address systemic deficiencies, transparent and need-based budgets must be implemented, suppliers must be paid continuously, PTC structures must be revitalised with clear authority and STG training at all levels. Strengthening medicine governance is not merely a technical undertaking, but also a fundamental requirement for the implementation of universal health insurance, which depends on equity, ethical practice, and accountability. By adopting systematic and coordinated reforms, the situation in East Cape and other similar contexts can be improved to move towards a more resilient and just pharmaceutical system that respects the constitutional right to health of all people.
